# The effectiveness of transcranial photobiomodulation therapy (tPBM) and transauricular vagus nerve stimulation (taVNS) on reducing alcohol craving

**DOI:** 10.1016/j.neurot.2026.e00937

**Published:** 2026-06-08

**Authors:** Manjae Kwon, Minkyung Park, Minjae Kim, Jungyoon Kang, Woo-Young Ahn, Dongil Chung, Jung-Seok Choi, Young-Chul Jung

**Affiliations:** a Institute of Behavioral Science in Medicine, Yonsei University College of Medicine, Seoul, South Korea; b Department of Psychiatry, Yonsei University College of Medicine, Seoul, South Korea; c Department of Psychiatry, Samsung Medical Center, Sungkyunkwan University School of Medicine, Seoul, South Korea; d Department of Biomedical Engineering, Ulsan National Institute of Science and Technology, Ulsan, South Korea; e Department of Psychology, Seoul National University, Seoul, South Korea; f Department of Brain and Cognitive Sciences, Seoul National University, Seoul, South Korea; g AI Institute, Seoul National University, Seoul, South Korea

**Keywords:** Alcohol, Craving, Transcranial photobiomodulation (tPBM), Transauricular vagus nerve stimulation (taVNS), Noninvasive brain stimulation

## Abstract

Alcohol craving plays a central role in sustaining alcohol use and contributing to relapse. Noninvasive neuromodulation techniques such as transcranial photobiomodulation (tPBM) and transauricular vagus nerve stimulation (taVNS) may target craving-related neural circuits through complementary cortical and subcortical mechanisms. However, their relative and combined effects on craving and related symptoms remain unclear. To compare the effects of tPBM, taVNS, and combined tPBM + taVNS on alcohol craving, alcohol use, and psychological outcomes in individuals with subclinical alcohol use. Sixty participants were randomized to one of three intervention groups: tPBM, taVNS, or combined tPBM + taVNS. Participants self-administered the assigned stimulation five times per week for five weeks using portable home devices (15 min/session, total 25 sessions). Change in craving was the primary outcome. Both tPBM and combined tPBM + taVNS produced significant reductions in craving (PACS: *p* < 0.001) and alcohol use (AUDIT: *p* ≤ 0.011) compared with taVNS alone. ANCOVA revealed a significant group effect on craving (*F*(2,54) = 5.24, *p* = 0.008). Logistic regression showed higher odds of craving response with tPBM (*OR* = 8.09) and of drinking-behavior improvement with combined stimulation (*OR* = 16.73). tPBM and combined tPBM + taVNS effectively reduced craving, alcohol use in individuals with subclinical alcohol use.

## Introduction

Alcohol craving is a central mechanism sustaining alcohol use and relapse [1]. It arises from dysregulation within prefrontal–limbic circuits governing reward, emotion, and self-control, leading to impaired inhibition and heightened salience of alcohol cues [2]. While pharmacological interventions remain the standard of care, their clinical utility is often limited by partial efficacy and adverse effects, leaving many individuals with persistent, unmanageable craving [3,4]. This highlights the critical need for novel therapeutic strategies that can directly modulate the neural substrates of addiction.

Noninvasive brain stimulation (NIBS) has emerged as a promising tool for modulating the dysfunctional neural circuits involved in addiction [5]. However, it remains unclear whether targeting cortical executive networks or subcortical autonomic pathways yields superior clinical outcomes in reducing craving. Transcranial photobiomodulation (tPBM) and transauricular vagus nerve stimulation (taVNS) represent two distinct mechanistic approaches to this challenge.

The rationale for combining these modalities lies in their potential to synergistically target the neural substrates of addiction [6]. tPBM delivers near-infrared light to cortical tissue, enhancing mitochondrial respiration and cerebral blood flow [7]. This “top-down” stimulation of the prefrontal cortex has been shown to improve cognitive performance and mood, suggesting it may restore the cortical control required to dampen craving [8,9]. Conversely, taVNS targets “bottom-up” pathways by stimulating vagus afferents that project to limbic and monoaminergic structures, such as the amygdala and locus coeruleus [10]. By modulating these subcortical networks, taVNS has demonstrated efficacy in alleviating depressive symptoms and improving sleep quality in alcohol dependent populations [11,12]. Therefore, simultaneous stimulation may offer a comprehensive approach by engaging both cortical executive control and subcortical emotional regulation networks [10,13].

The present study aimed to directly compare the effects of these two distinct mechanistic approaches – cortical stimulation (tPBM), vagal stimulation (taVNS), and their combination – on alcohol craving in individuals with subclinical alcohol use. Specifically, we investigated the efficacy of 5-week intervention protocol. We hypothesized that tPBM and taVNS would reduce alcohol craving through distinct top-down cortical and bottom-up vagal pathways, respectively, and that their combined application would yield the greatest reduction in craving by addressing the multifaceted nature of addiction circuitry.

## Materials and methods

### Participants

Participants were recruited through online advertisements. All participants provided written informed consent prior to participation, and all procedures were approved by the Institutional Review Board of Yonsei University Health System and Samsung Medical Center, in accordance with the Declaration of Helsinki.

Eligible participants were adults aged between 19 and 40 years who were capable of providing valid informed consent. All participants were current alcohol drinkers with a score of 4 or higher on the Korean version of the Alcohol Use Disorders Identification Test (AUDIT-K) [14], but did not meet the diagnostic criteria for Alcohol Use Disorder (AUD) according to the Diagnostic and Statistical Manual of Mental Disorders, Fifth Edition (DSM-5) [15]. Additional inclusion criteria required that participants were able to complete the stimulation program over a five-week period and voluntarily agreed to participate by providing written informed consent.

Exclusion criteria included inability to read or comprehend the consent form (e.g., due to illiteracy or non-Korean language background) or lack of capacity for independent decision-making. Pregnant women and individuals considered vulnerable—such as employees, students affiliated with the research institution, investigators, or study sponsor—were also excluded. Participants were further excluded if they were unable to complete the five-week stimulation program, met DSM-5 criteria for Alcohol Use Disorder, had a substance use disorder involving substances other than alcohol or nicotine, or reported a metal allergy that could interfere with electroencephalography (EEG) recording or stimulation procedures.

**Fig. 1:**
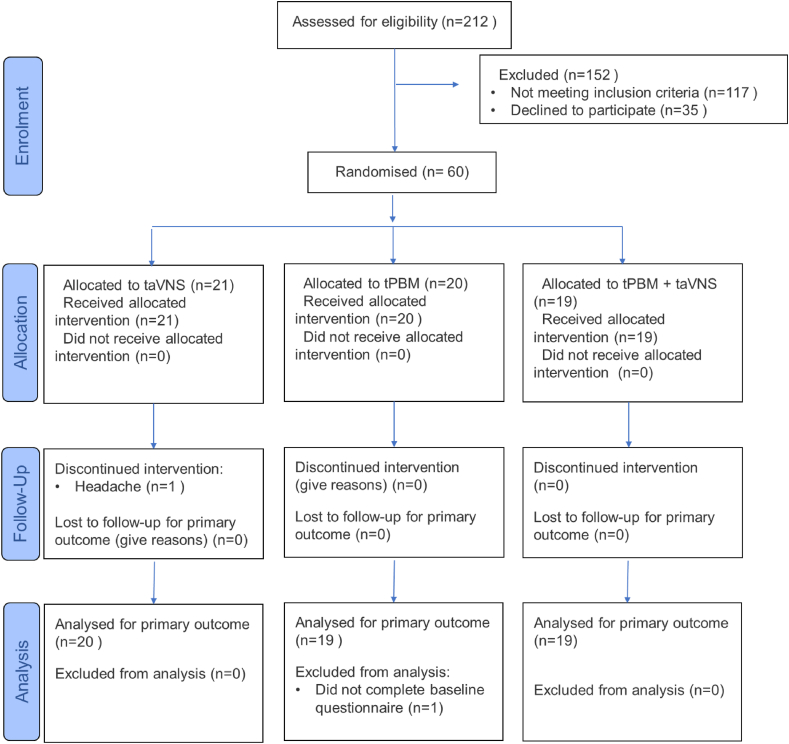
CONSORT Flow diagram.

A total of 60 participants were enrolled, of whom 59 completed the full five-week intervention and 58 were included in the final analysis (Fig. 1).

### Procedure and stimulation protocol

This study employed a parallel, open-label, randomized, uncontrolled design with three intervention arms: taVNS, tPBM, and combined taVNS + tPBM. A total of sixty participants were randomly allocated in equal numbers to one of the three groups. Each participant underwent pre- and post-intervention assessments conducted within one week before and after the five-week treatment period. This trial was registered at ClinicalTrials.gov (Identifier: NCT07071493).

Participants self-administered the assigned intervention five times per week for five consecutive weeks (15 min per session, total 25 sessions) under remote supervision. Adherence and safety were monitored through electronic device logs and weekly check-ins by research staff. Participants were instructed to refrain from alcohol consumption for at least 24 h before each stimulation session.

For the taVNS condition, stimulation was delivered using the Healaon PRO device (Neurive Inc., Seoul, Republic of Korea). taVNS parameters were selected in accordance with the international consensus reporting standards for transcutaneous vagus nerve stimulation [16]. Electrodes were placed bilaterally at cymba concha and cavum concha, which is innervated by the auricular branch of the vagus nerve [17,18], and simultaneous stimulation of both subregions evokes larger vagal somatosensory evoked potentials [19]. Bilateral placement was chosen to increase afferent recruitment, as transcutaneous current is constrained by sensory discomfort at higher amplitudes [20]. The 30 Hz frequency, 200 μs pulse width, and 15-min session duration fall within the most frequently reported and empirically supported ranges for taVNS [21] and correspond to a protocol that produces measurable autonomic engagement in adults [22,23] and improvements in mood, sleep, and autonomic balance during sustained daily home use [24]. Stimulation intensity was titrated individually to a comfortable, clearly perceptible sensation (0.5–2.5 mA). For the tPBM condition, near-infrared light was delivered transcranially using the iSyncMe device (iMediSync Inc., Seoul, Republic of Korea). The device's LED bulbs delivered light with a wavelength of 850 nm to 19 channels across the scalp, corresponding to locations in the international 10–20 system. The 15-min session consisted of three sequential 5-min phases: 1) parietal and occipital stimulation at a 10 Hz pulse, 2) whole-brain stimulation at a 15 Hz pulse, and 3) frontal stimulation at an 18 Hz pulse. Stimulation regions were selected to correspond to cortical areas that show reproducible functional and aberrant oscillatory activity in heavy alcohol use and AUD. Effects of tPBM on specific oscillatory bands have been reported but are not yet systematically characterized, so regions were targeted on the basis of their documented abnormality rather than a presumed band-specific effect. Parietal and occipital cortex was targeted because heavy drinkers and patients with AUD show blunted occipital alpha during visual processing [25,26] and reduced resting alpha power [27], with the degree of reduction tracking severity. The intervening whole-scalp phase addressed the widespread cortical disturbance reported in AUD, including elevation of beta-band activity [28,29]. Frontal cortex was targeted to support the prefrontal top-down control over reward-driven behavior that is impaired in addiction [30].

In the combined condition, taVNS and tPBM were administered simultaneously, following the same parameters as in the single-modality interventions. Participants received detailed training in device operation, safety, and reporting procedures during the initial laboratory session. Compliance was verified throughout the intervention period using automated usage tracking and weekly contact. Adverse events were monitored through phone consultation once a week with the researcher. No participant reported serious adverse effects; one participant discontinued the study due to dizziness following the combined use of PBM and VNS.

### Assessments and outcome measures

All outcome measures were assessed at baseline and after completion of the five-week intervention period. The primary outcome was the change in alcohol craving, as measured by the Penn Alcohol Craving Scale (PACS) [31]. Secondary outcomes included psychometric instruments assessing alcohol use, affective symptoms, and other psychological domains. Alcohol use behavior was assessed using the Alcohol Use Disorders Identification Test (AUDIT) [14,32]. Depressive and anxiety symptoms were evaluated using the Beck Depression Inventory (BDI) [33,34] and Beck Anxiety Inventory (BAI) [35], respectively. Sleep quality was measured with the Pittsburgh Sleep Quality Index (PSQI) [36], and impulsivity was assessed using the Barratt Impulsiveness Scale (BIS-11) [37].

### Statistical analysis

All statistical analyses were performed using R version 4.2.2. Statistical significance was set at *p* < 0.05 (two-tailed). Within-group changes from baseline to post-intervention were analyzed using paired two-sample *t*-tests for all continuous outcome measures. Between-group differences in post-intervention scores were examined using analysis of covariance (ANCOVA), with baseline scores included as covariates to control for initial group differences.

Treatment response was defined as a ≥30% reduction from baseline in PACS or AUDIT scores at week 5. No empirically validated responder threshold has been established for either instrument. The 30% cutoff was selected in line with conventions used in adjacent psychiatric trial literatures: a ≥30% reduction in the Positive and Negative Syndrome Scale (PANSS) is widely used to define treatment response in schizophrenia [38], and reductions in the 25–35% range on the Yale-Brown Obsessive Compulsive Scale (Y-BOCS) have been identified as the most predictive range for treatment response in obsessive-compulsive disorder [[39], [40], [41]]. To assess the robustness of the responder findings to threshold choice, we additionally conducted a sensitivity analysis at 20% and 50% reduction thresholds; full results are reported in Supplementary Fig. S1 and Supplementary Table S1. To identify predictors of treatment efficacy, multiple linear regression analyses were performed with changes in PACS and AUDIT scores as dependent variables. Baseline demographic and psychological measures (including age, sex, BDI, BAI scores) were entered as independent variables to assess their potential contribution to individual variability in treatment response. All data were tested for normality using the Shapiro–Wilk test, and model assumptions were verified. When appropriate, effect sizes (Cohen's *d* for *t*-tests and partial *η*^*2*^ for ANCOVA) were reported to indicate the magnitude of observed effects.

## Results

### Participant characteristics

A total of 60 participants were enrolled and randomly allocated to one of three intervention groups: taVNS (*n* = 20), tPBM (*n* = 20), and combined taVNS + tPBM (*n* = 20). 58 participants completed 5-week intervention and completed both baseline and post-intervention assessments and were included in the analyses. Baseline demographic and clinical characteristics did not differ significantly among the three groups (Table 1).

**Table 1:** Demographic and baseline clinical characteristics of participants by intervention group.

	taVNS (*n* = 20)	tPBM (*n* = 19)	tPBM + taVNS (*n* = 19)	*p*-value
age (mean (SD))	26.45 (4.51)	28.53 (4.89)	27.63 (4.79)	0.39
Sex (%)				
Male	9 (45.0)	8 (42.10)	8 (42.1)	0.98
Female	11 (55.0)	11 (57.90)	11 (57.9)	
Marriage (%)				
Unmarried	18 (90.0)	17 (89.50)	16 (84.2)	0.83
Married	2 (10.0)	2 (10.50)	3 (15.8)	
Education (years, mean (SD))	15.65 (1.42)	15.84 (1.95)	16.21 (1.99)	0.62
AUDIT (mean (SD))	10.55 (6.16)	10.58 (5.01)	10.37 (5.20)	0.99
PACS (mean (SD))	7.95 (5.34)	7.32 (4.71)	6.42 (2.67)	0.56
BDI (mean (SD))	9.30 (6.78)	8.68 (7.79)	8.47 (4.93)	0.92
BAI (mean (SD))	3.95 (4.95)	4.26 (4.92)	3.00 (3.07)	0.66
BIS. (mean (SD))	64.45 (11.03)	62.58 (11.01)	61.32 (9.86)	0.65
PSQI (mean (SD))	6.60 (2.33)	6.95 (2.80)	5.42 (2.09)	0.14

**Abbreviations:** AUDIT, Alcohol Use Disorders Identification Test; PACS, Penn Alcohol Craving Scale; BDI, Beck Depression Inventory; BAI, Beck Anxiety Inventory; BIS, Barratt Impulsiveness Scale; PSQI, Pittsburgh Sleep Quality Index; taVNS, transauricular vagus nerve stimulation; tPBM, transcranial photobiomodulation.

### Primary outcome

Within-group analyses revealed significant reductions in PACS scores from baseline to post-intervention in both the tPBM and combined taVNS + tPBM groups (paired t-tests: tPBM, *t*(18) = 5.96, *p* < 0.001; combined, *t*(18) = 5.08, *p* < 0.001; Table 2), whereas the taVNS-only group did not show a significant change (*t*(19) = −1.56, *p* = 0.14).

**Table 2:** Within-group pre–post changes in outcome measures from baseline to week 5.

Outcome	Group	Baseline Mean (SD)	Week 5 Mean (SD)	Mean Difference (SD)	*t*-score	*p*-value	Effect size (*d*)
Craving (PACS)	**taVNS**	7.95 (5.34)	6.55 (4.50)	−1.40 (4.02)	−1.56	0.136	0.35
	**tPBM**	7.32 (4.71)	4.11 (3.94)	−3.21 (2.35)	5.96	<0.001∗	1.37
	**tPBM + taVNS**	6.42 (2.67)	2.89 (2.66)	−3.53 (3.03)	5.08	<0.001∗	1.17
AUDIT	**taVNS**	10.55 (6.16)	9.40 (5.14)	−1.15 (4.28)	1.2	0.245	0.27
	**tPBM**	10.58 (5.01)	8.11 (4.86)	−2.47 (2.82)	3.83	0.001∗	0.88
	**tPBM + taVNS**	10.37 (5.20)	7.26 (3.21)	−3.11 (4.77)	2.84	0.011∗	0.65
Depression (BDI)	**taVNS**	9.30 (6.78)	7.60 (4.89)	−1.70 (6.03)	1.26	0.222	0.28
	**tPBM**	8.68 (7.79)	5.79 (8.29)	−2.89 (5.92)	2.13	0.047∗	0.49
	**tPBM + taVNS**	8.47 (4.93)	5.16 (4.37)	−3.32 (5.19)	2.79	0.012∗	0.64
Anxiety (Bai)	**taVNS**	3.95 (4.95)	3.40 (5.44)	−0.55 (4.36)	0.56	0.579	0.13
	**tPBM**	4.26 (4.92)	5.37 (6.59)	1.11 (3.13)	−1.54	0.141	−0.35
	**tPBM + taVNS**	3.00 (3.07)	3.00 (3.43)	0.00 (2.85)	0	1.000	0
Sleep (PSQI)	**taVNS**	6.60 (2.33)	5.90 (2.45)	−0.70 (3.29)	0.95	0.354	0.21
	**tPBM**	6.95 (2.80)	6.42 (2.83)	−0.53 (2.50)	0.92	0.371	0.21
	**tPBM + taVNS**	5.42 (2.09)	5.42 (2.04)	0.00 (2.03)	0	1	0

Values are presented as mean (standard deviation). Paired t-tests were performed to compare pre- and post-intervention scores within each group. *p* < 0.05 was considered statistically significant. **Abbreviations:** PACS, Penn Alcohol Craving Scale; AUDIT, Alcohol Use Disorders Identification Test; BDI, Beck Depression Inventory; BAI, Beck Anxiety Inventory; PSQI, Pittsburgh Sleep Quality Index; taVNS, transauricular vagus nerve stimulation; tPBM, transcranial photobiomodulation.

**Fig. 2:**
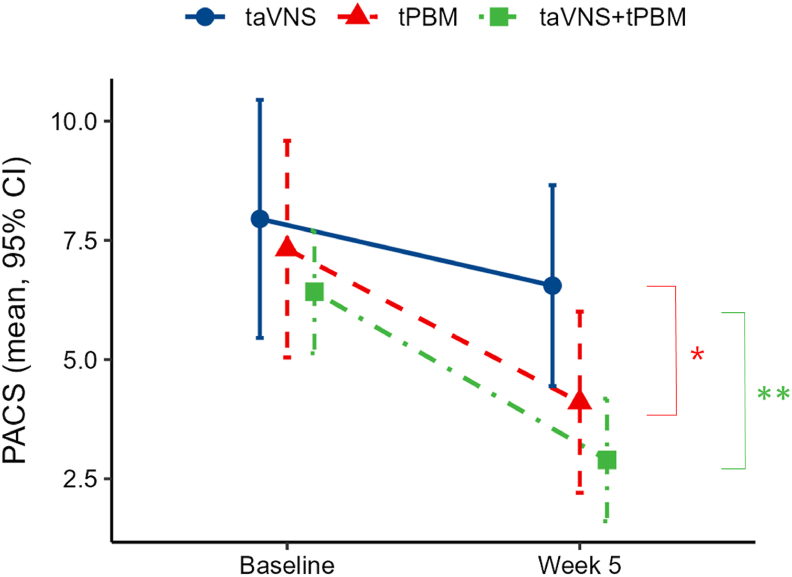
Changes in alcohol craving across intervention groups over 5 weeks. Mean Penn Alcohol Craving Scale (PACS) scores (±95% CI) are shown for each group at baseline and week 5. ANCOVA controlling for baseline PACS scores revealed a significant main effect of group on post-intervention craving levels (*F*(2, 54) = 5.24, *p* = 0.008). Post-hoc pairwise comparisons with Holm adjustment indicated that both the tPBM (*p* = 0.045) and combined taVNS + tPBM (*p* = 0.001) groups showed significantly greater reductions in craving compared with the taVNS-only group, whereas the difference between the two tPBM-containing groups was not significant (*p* = 0.45).

ANCOVA controlling for baseline PACS scores revealed a significant main effect of group on post-intervention craving levels (*F*(2, 54) = 5.24, *p* = 0.008; Table 3, Fig. 2). Post-hoc pairwise comparisons with Holm correction indicated that both the tPBM (*p* = 0.045, Holm-adjusted) and combined taVNS + tPBM (*p* = 0.001, Holm-adjusted) groups showed significantly greater reductions in craving compared with the taVNS-only group, while the difference between the tPBM and combined groups was not significant (*p* = 0.45, Holm-adjusted**).**

**Table 3:** ANCOVA results comparing post-intervention outcomes across intervention groups, controlling for baseline scores.

Outcome	*F* (2,54)	*p*-value	Partial *η*^*2*^
Craving	5.24	**0.008∗∗**	0.16
AUDIT	1.92	0.16	0.07
Depression	0.86	0.43	0.03
Anxiety	1.17	0.32	0.04
Sleep	0.17	0.84	0.006

∗Descriptive statistics and within-group paired t-tests are reported in Table 2. Double asterisks denote statistical significance (p < 0.01).

**Abbreviations:** PACS, Penn Alcohol Craving Scale; AUDIT, Alcohol Use Disorders Identification Test; BDI, Beck Depression Inventory; BAI, Beck Anxiety Inventory; PSQI, Pittsburgh Sleep Quality Index; taVNS, transauricular vagus nerve stimulation; tPBM, transcranial photobiomodulation.

### Secondary outcomes

Significant within-group improvements were observed in alcohol use severity (AUDIT) and depressive symptoms (BDI) in the tPBM and combined taVNS + tPBM groups (paired t-tests: AUDIT—tPBM, *t*(18) = 3.83, *p* = 0.001; combined, *t*(18) = 2.84, *p* = 0.011; BDI—tPBM, *t*(18) = 2.13, *p* = 0.047; combined, *t*(18) = 2.79, *p* = 0.012), whereas the taVNS-only group did not show significant change in either measure (*t*(19) = 1.20, *p* = 0.25, *t*(19) = 1.26, *p* = 0.22, respectively).

ANCOVA analyses for secondary outcomes revealed no significant group effects for alcohol habit (AUDIT), depression (BDI), anxiety (BAI), sleep quality (PSQI) (Table 3).

Correlation analysis showed a positive association between changes in craving (PACS) and changes in depressive symptoms (BDI), although this did not reach statistical significance (*r* = 0.24, *p* = 0.067).

**Fig. 3:**
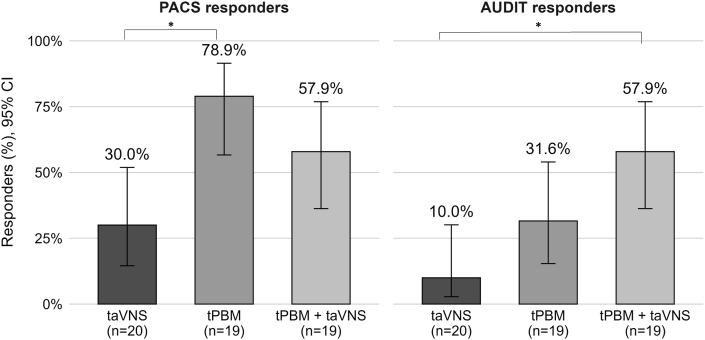
Response rates (≥30% reduction from baseline) in alcohol craving and alcohol use across intervention groups. Bars represent the percentage of responders (mean ± 95% confidence interval) for each treatment condition: transauricular vagus nerve stimulation (taVNS), transcranial photobiomodulation (tPBM), and combined taVNS + tPBM. Response was defined as a ≥30% reduction from baseline on the Penn Alcohol Craving Scale (PACS) or Alcohol Use Disorders Identification Test (AUDIT) at week 5. Logistic regression analyses adjusted for age, sex, and baseline scores indicated that the tPBM group showed significantly higher odds of PACS response compared with the taVNS group (*p* = 0.006), and the combined tPBM + taVNS group showed higher odds of AUDIT response compared with the taVNS group (*p* = 0.003). Asterisks indicate significant between-group differences based on adjusted logistic regression (*p* < 0.05).

Logistic regression analyses examined predictors of treatment response. For the PACS response, the tPBM-only group showed significantly higher odds compared with the taVNS-only group (*OR* = 8.75, 95% CI = 2.07–37.05, *p* = 0.004; Table 4, Fig. 3), which was significant after adjusting for age, sex, baseline PACS score (*OR* = 8.09, 95% CI = 1.81–36.10, *p* = 0.006). For the AUDIT response (≥30% reduction from baseline), the combined taVNS + tPBM group demonstrated significantly higher odds of achieving a response compared with the taVNS-only group (*OR* = 12.37, 95% CI = 2.59–93.14, *p* = 0.004), which was significant after adjusting for age, sex, baseline AUDIT score (*OR* = 16.73, 95% CI = 3.16–144.45, *p* = 0.003). Sensitivity analyses using 20% and 50% reduction thresholds yielded consistent findings: tPBM and combined tPBM + taVNS produced higher responder rates than taVNS at every threshold for both PACS and AUDIT (Supplementary Fig. S1, Supplementary Table S1). Significant adjusted between-group differences were observed at multiple thresholds, including for tPBM versus taVNS on PACS at 20% (*OR =* 32.74, *p* = 0.002) and for combined tPBM + taVNS versus taVNS on AUDIT at 50% (*OR* = 12.15, *p* = 0.041).

**Table 4:** Response rates (≥30% reduction from baseline) at week 5.

Outcome	Group	Responders *n/N* (%)	Unadjusted odds ratio (95% CI)	p-value	Adjusted odds ratio^a^ (95% CI)	*p*-value
PACS (craving)	**taVNS**	6/20 (30.0%)	Ref.	–	Ref.	–
	**tPBM**	15/19 (78.9%)	8.75 (2.07–37.05)	0.004∗	8.09 (1.81–36.10)	0.006∗
	**tPBM + taVNS**	11/19 (57.9%)	3.21 (0.88–12.71)	0.084	3.03 (0.80–12.50)	0.111
AUDIT (alcohol use)	**taVNS**	2/20 (10.0%)	Ref.	–	Ref.	–
	**tPBM**	6/19 (31.6%)	4.15 (0.81–31.58)	0.11	5.25 (0.94–43.34)	0.078
	**tPBM + taVNS**	11/19 (57.9%)	12.37 (2.59–93.14)	0.004∗∗	16.73 (3.16–144.35)	0.003∗

∗Asterisks denote statistical significance (*p* < 0.05; *p* < 0.01).

**Abbreviations**: PACS, Penn Alcohol Craving Scale; AUDIT, Alcohol Use Disorders Identification Test; taVNS, transauricular vagus nerve stimulation; tPBM, transcranial photobiomodulation; OR, odds ratio; CI, confidence interval.

a adjusted for age, sex, baseline PACS (for craving), baseline AUDIT score (for alcohol use).

**Fig. 4:**
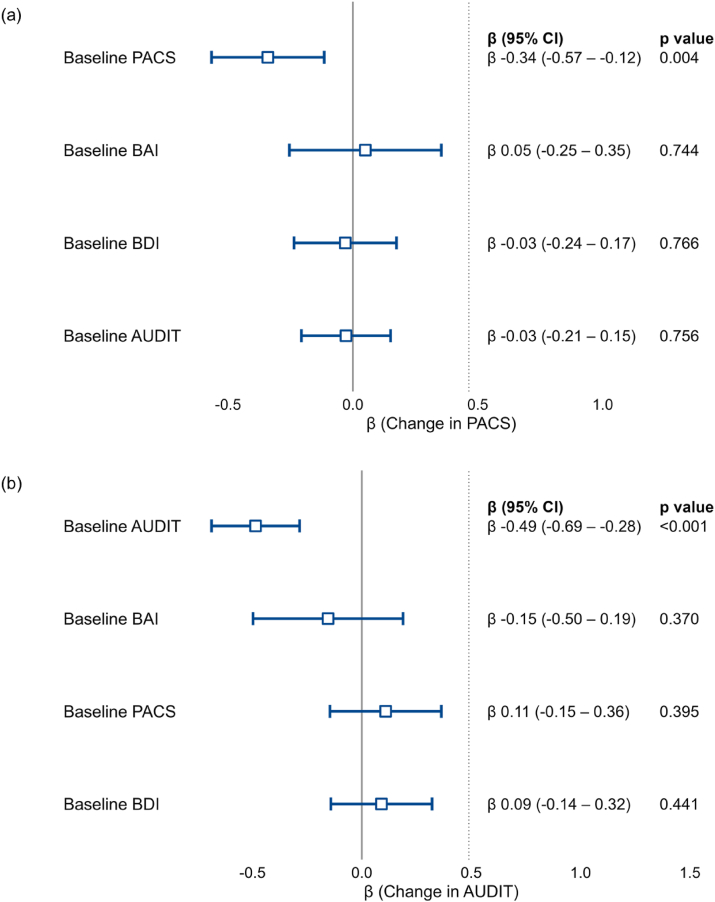
Forest plots showing standardized regression coefficients (*β*) and 95% confidence intervals (CI) for predictors of change in (a) Penn Alcohol Craving Scale (ΔPACS) and (b) Alcohol Use Disorders Identification Test (ΔAUDIT) scores following 5 weeks of intervention. Multiple linear regression analyses included baseline variables as predictors: AUDIT, PACS, Beck Depression Inventory (BDI), and Beck Anxiety Inventory (BAI) scores. Higher baseline PACS scores significantly predicted greater reduction in craving (ΔPACS; *β* = −0.34, *p* = 0.004), and higher baseline AUDIT scores significantly predicted greater reduction in alcohol use (ΔAUDIT; *β* = −0.49, *p* < 0.001). Error bars represent 95% confidence intervals. **Abbreviations:** PACS, Penn Alcohol Craving Scale; AUDIT, Alcohol Use Disorders Identification Test; BDI, Beck Depression Inventory; BAI, Beck Anxiety Inventory; CI, confidence interval.

Linear regression analyses revealed that baseline PACS scores significantly predicted the magnitude of craving reduction (ΔPACS; *β* = [−0.34], *p* = 0.004; Fig. 4a), and baseline AUDIT scores significantly predicted the change in AUDIT scores (ΔAUDIT; *β* = [−0.49], *p* < 0.001; Fig. 4b), indicating that higher initial severity was associated with greater improvement.

## Discussion

This study is the first to directly compare and combine the efficacy of tPBM and taVNS neuromodulation for reducing alcohol craving. Our primary finding is that a five-week course of tPBM, administered alone or in combination with taVNS, resulted in a significant reduction in alcohol craving. In contrast, taVNS alone did not produce a significant change. These results partially support our hypothesis, suggesting that neuromodulation targeting the frontal cortical network is a potent strategy for craving reduction, while the incremental benefit of concurrently stimulating vagal afferent pathways remains limited in this specific population.

The marked efficacy of tPBM in reducing alcohol craving aligns with its proposed mechanism of enhancing prefrontal cortical function. Addiction is widely conceptualized as a disorder of impaired prefrontal top-down control over subcortical reward-driven behaviors [30]. Our tPBM protocol employed a multi-phase sequence concluding with a focused stimulation of the frontal region at 18 Hz, targeting frontal networks with beta-band frequency, which is associated with alertness and cognitive control [42]. tPBM acts by stimulating mitochondrial cytochrome-*c*-oxidase and boosting ATP production [13,43]. Our findings suggest that this enhancement of prefrontal resources bolsters the cognitive control circuits crucial for inhibiting prepotent responses to alcohol cues. This extends previous research on prefrontal tPBM for opioid craving [8] and positions it as a promising non-pharmacological intervention for a broader range of substance use issues.

Conversely, the lack of a significant effect of taVNS on craving may be attributed to the characteristics of our study population rather than the inefficacy of the intervention itself. Previous studies demonstrating the benefits of taVNS primarily focused on patients with alcohol dependence experiencing protracted withdrawal symptoms, such as anxiety and autonomic instability [12]. taVNS targets these symptoms via “bottom-up” modulation of the nucleus of the solitary tract (NTS) and noradrenergic projections [44]. However, our sample consisted of individuals with subclinical alcohol use who likely lacked significant withdrawal symptoms or severe autonomic dysregulation. Consequently, the therapeutic target of taVNS, the alleviation of negative affect and withdrawal distress, was largely absent in this group, potentially explaining the result.

Contrary to our initial hypothesis, the combined tPBM + taVNS intervention did not produce a significantly greater reduction in craving compared to tPBM alone. This may suggest a ceiling effect, whereby the robust impact of tPBM was the primary driver of craving reduction. However, a nuance emerged from our responder analysis: while the tPBM group had the highest odds of a craving reduction, the combined group showed the highest odds of a reduction in overall problematic alcohol use as assessed by AUDIT. This discrepancy implies a dissociation between the urge to drink and drinking behavior. It is possible that while tPBM strengthens cognitive resistance to urges, the addition of taVNS may subtly modulate broader behavioral patterns, leading to changes in actual consumption. Notably, the positive correlation between baseline severity and the magnitude of improvement suggests that the therapeutic effects might be even more pronounced in a clinical population. This provides a strong rationale for future efficacy trials in patients with AUD diagnosis.

This study also demonstrated the high feasibility of tPBM and taVNS neuromodulation. Participants achieved a compliance rate of 98%, which compares favorably to typical attrition rate in outpatient addiction programs, which often range from 30% to 50% [45,46]. This confirms that such neuromodulation devices can effectively bridge the gap between clinical efficacy and real-world accessibility.

However, the findings must be interpreted in light of several significant limitations. This study uses open-label, uncontrolled design, which lacks a sham condition. Adding a credible sham control may help determine true efficacy. Although the lack of effect in the taVNS group argues against a purely non-specific effect, a rigorous, double-blind, sham-controlled trial is the necessary next step to validate these results. Second, the focus in a subclinical population limits generalization to individuals with diagnosed AUD, who may exhibit different neural responsiveness to the interventions. Third, the five-week duration may have been insufficient to elicit the full therapeutic potential, particularly for taVNS, which can have slower-acting cumulative effects [47]. Finally, no dose-response relationship has been established for taVNS in craving or alcohol-related outcomes, and the stimulation parameters used here should be regarded as physiologically validated and tolerable values drawn from an empirically supported range rather than as settings optimized for craving reduction; systematic dose-finding in alcohol-using populations remains needed.

In conclusion, this investigation highlights tPBM as an effective intervention for managing alcohol craving. Future research should prioritize large-scale, randomized, sham-controlled trials in clinical populations with diagnosed AUD. Such studies should aim to determine whether the combination of cortical and vagal stimulation offers synergistic benefits for clinical population.

## CRediT authorship contribution statemenent

**Manjae Kwon:** Writing-original draft, Conceptualization, Methodology, Formal analysis. **Minkyung Park**: Writing-review and editing, Formal analysis, Data curation, Methodology, Project administration, **Minjae Kim:** Writing-review and editing, Investigation, Formal analysis, Data curation, Project administration, **Jungyoon Kang:** Investigation, Validation**,** Project administration, **Woo-Young Ahn:** Writing-review and editing, Supervision, Conceptualization, Methodology, Funding acquisition, **Dongil-Chung:** Writing-review and editing, Supervision, Conceptualization, Methodology, Resources, Investigation, Funding acquisition, **Jung-Seok Choi:** Writing-review and editing, Supervision, Conceptualization, Methodology, Resources, Investigation, Funding acquisition, **Young-Chul Jung:** Writing-review and editing, Supervision, Conceptualization, Methodology, Resources, Investigation, Funding acquisition.

## Declaration of competing interest

The authors declare that they have no known competing financial interests or personal relationships that could have appeared to influence the work reported in this paper.
