# Supplementary material for: The effectiveness of transcranial photobiomodulation therapy (tPBM) and transauricular vagus nerve stimulation (taVNS) on reducing alcohol craving
Source: Neurotherapeutics. 2026 Jun 8;23(4):e00937. doi: 10.1016/j.neurot.2026.e00937 (PMC13264161; doi:10.1016/j.neurot.2026.e00937)
Supplement: Multimedia component 1 [file mmc1.docx]

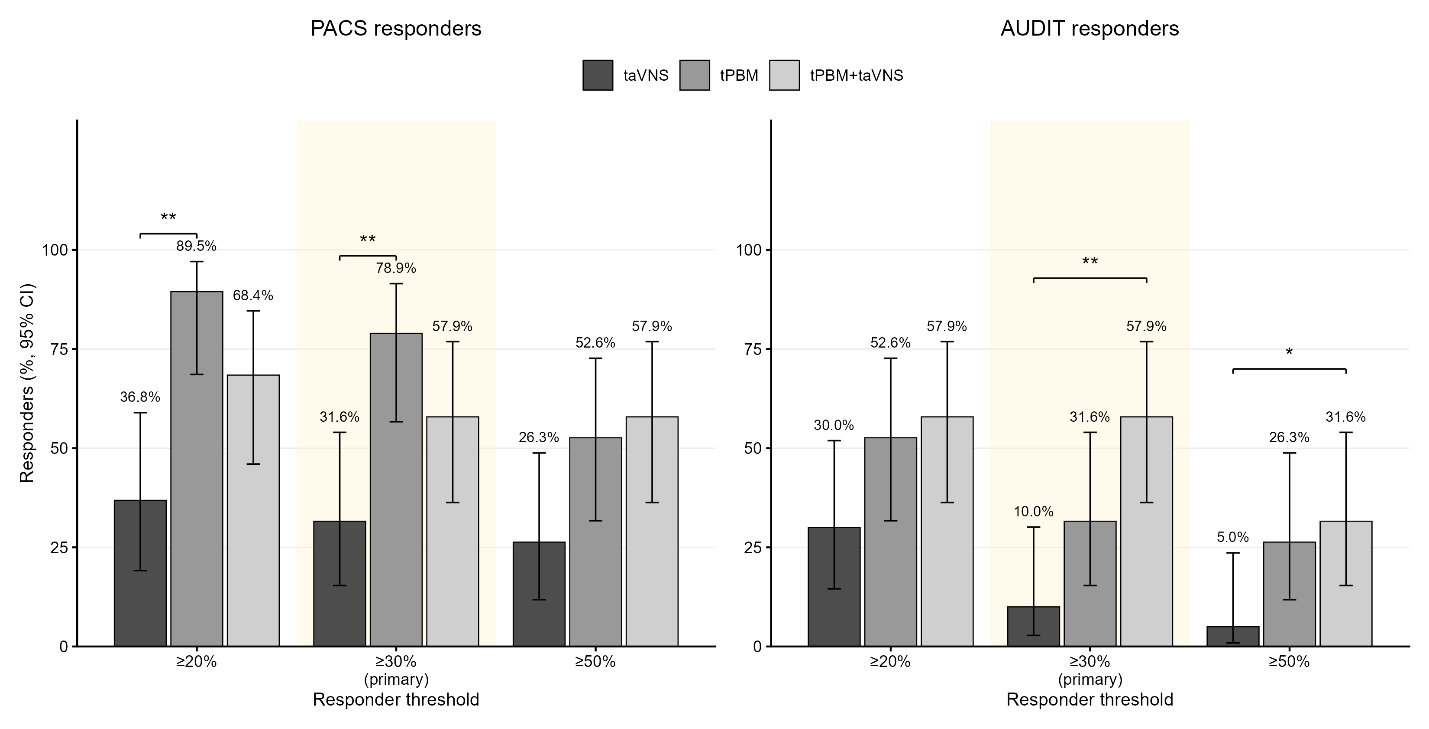


**Supplementary Fig. S1** Sensitivity analysis for AUDIT and PACS response. Responder rates (%) for the Penn Alcohol Craving Scale (PACS) and the Alcohol Use Disorders Identification Test (AUDIT) at three thresholds (≥20%, ≥30%, and ≥50% reduction from baseline at week 5) across the three intervention groups. The number of responders over the number analysed (n/N) is shown above each bar. The ≥30% threshold corresponds to the primary responder analysis reported in the main text. Between-group odds ratios at each threshold are given in Supplementary Table S1.

**Supplementary Table S1.** Sensitivity of responder analysis to threshold choice.

| **Threshold** | **Group** | **Responders, n/N (%)** | **Unadjusted OR (95% CI), p** | **Adjusted OR (95% CI), p** |
| --- | --- | --- | --- | --- |
| ***PACS (Penn Alcohol Craving Scale)*** | | | | |
| ≥20% | taVNS | 7/20 (35.0) | Ref. | Ref. |
|  | tPBM | 17/19 (89.5) | 15.79 (2.81–88.65), 0.002 | 32.74 (3.74–286.50), 0.002 |
|  | tPBM + taVNS | 13/19 (68.4) | 4.02 (1.06–15.30), 0.041 | 4.36 (1.04–18.32), 0.044 |
| ≥30% | taVNS | 6/20 (30.0) | Ref. | Ref. |
|  | tPBM | 15/19 (78.9) | 8.75 (2.07–37.05), 0.004 | 8.09 (1.81–36.10), 0.006 |
|  | tPBM + taVNS | 11/19 (57.9) | 3.21 (0.85–12.07), 0.084 | 3.06 (0.79–11.88), 0.107 |
| ≥50% | taVNS | 5/20 (25.0) | Ref. | Ref. |
|  | tPBM | 10/19 (52.6) | 3.33 (0.85–13.03), 0.082 | 3.04 (0.76–12.12), 0.115 |
|  | tPBM + taVNS | 11/19 (57.9) | 4.13 (1.05–16.18), 0.041 | 3.83 (0.96–15.27), 0.057 |
| ***AUDIT (Alcohol Use Disorders Identification Test)*** | | | | |
| ≥20% | taVNS | 6/20 (30.0) | Ref. | Ref. |
|  | tPBM | 10/19 (52.6) | 2.59 (0.69–9.71), 0.155 | 3.05 (0.77–12.13), 0.113 |
|  | tPBM + taVNS | 11/19 (57.9) | 3.21 (0.85–12.07), 0.084 | 3.63 (0.92–14.35), 0.066 |
| ≥30% | taVNS | 2/20 (10.0) | Ref. | Ref. |
|  | tPBM | 6/19 (31.6) | 4.15 (0.71–24.05), 0.111 | 5.25 (0.83–33.06), 0.078 |
|  | tPBM + taVNS | 11/19 (57.9) | 12.37 (2.21–69.27), 0.004 | 16.73 (2.63–106.39), 0.003 |
| ≥50% | taVNS | 1/20 (5.0) | Ref. | Ref. |
|  | tPBM | 5/19 (26.3) | 6.79 (0.71–65.20), 0.096 | 8.81 (0.81–95.78), 0.074 |
|  | tPBM + taVNS | 6/19 (31.6) | 8.77 (0.94–82.07), 0.057 | 12.15 (1.11–132.71), 0.041 |

*Note.* n/N, number of responders / total participants analysed; CI, confidence interval; OR, odds ratio; Ref., reference category. Unadjusted odds ratios were estimated by logistic regression with treatment group as the only predictor. Adjusted odds ratios were estimated by logistic regression including treatment group, age, sex, and the relevant baseline score (PACS or AUDIT) as covariates, with taVNS as the reference category. The ≥30% threshold corresponds to the primary analysis reported
